# Comparison of QEEG Findings before and after Onset of Post-COVID-19 Brain Fog Symptoms

**DOI:** 10.3390/s22176606

**Published:** 2022-09-01

**Authors:** Marta Kopańska, Danuta Ochojska, Renata Muchacka, Agnieszka Dejnowicz-Velitchkov, Agnieszka Banaś-Ząbczyk, Jacek Szczygielski

**Affiliations:** 1 Department of Pathophysiology, University of Rzeszow, 35-959 Rzeszow, Poland; 2 Department of Psychology, University of Rzeszow, 35-959 Rzeszow, Poland; 3 Department of Animal Physiology and Toxicology, Pedagogical University of Cracow of the National Education Commission, 30-084 Cracow, Poland; 4 ADEA Co., Ltd., Biofeedback Center, 1000 Sofia, Bulgaria; 5 Department of Biology, University of Rzeszow, 35-959 Rzeszow, Poland; 6 Faculty of Medicine, University of Rzeszow, 35-959 Rzeszow, Poland; 7 Department of Neurosurgery, Faculty of Medicine, Saarland University, 66421 Homburg, Germany

**Keywords:** QEEG, brain fog, COVID-19, patients, quantitative electroencephalography

## Abstract

Previous research and clinical reports have shown that some individuals after COVID-19 infection may demonstrate symptoms of so-called brain fog, manifested by cognitive impairment and disorganization in behavior. Meanwhile, in several other conditions, related to intellectual function, a specific pattern of changes in electric brain activity, as recorded by quantitative electroencephalography (QEEG) has been documented. We hypothesized, that in post-COVID brain fog, the subjective complaints may be accompanied by objective changes in the QEEG profile. In order to test this hypothesis, we have performed an exploratory study on the academic staff of our University with previous records of QEEG originating in the pre-COVID-19 era. Among them, 20 subjects who revealed neurological problems in the cognitive sphere (confirmed as covid fog/brain fog by a clinical specialist) after COVID-19 infection were identified. In those individuals, QEEG was performed. We observed, that opposite to baseline QEEG records, increased Theta and Alpha activity, as well as more intensive sensimotor rhythm (SMR) in C4 (right hemisphere) in relation to C3 (left hemisphere). Moreover, a visible increase in Beta 2 in relation to SMR in both hemispheres could be documented. Summarizing, we could demonstrate a clear change in QEEG activity patterns in individuals previously not affected by COVID-19 and now suffering from post-COVID-19 brain fog. These preliminary results warrant further interest in delineating their background. Here, both neuroinflammation and psychological stress, related to Sars-CoV2-infection may be considered. Based on our observation, the relevance of QEEG examination as a supportive tool for post-COVID clinical workup and for monitoring the treatment effects is also to be explored.

## 1. Introduction

The late and persistent consequences of COVID-19 infection are becoming a growing problem for the population worldwide. Many previous COVID-19 patients, despite the end of infection, indicate the occurrence of various ailments. Data obtained from the stop-covid.pl registration platform [1] (the first Polish program for the assessment of complications after COVID-19 in people who have not been hospitalized due to coronavirus) indicate that 10–20% of them still have pulmonary and cardiological complications. However, as much as 45% of them reported chronic fatigue. However, the most serious implications of the coronavirus infection are reported for mental health [2,3,4,5,6].

Here, some non-specific problems with remembering (short-term memory) and recalling certain facts (long-term memory) and also in spatial orientation are most commonly reported. They are accompanied by difficulties in concentrating attention and associating and concluding (thinking), sensitivity to light and sound, and a feeling of chronic fatigue [7,8].

Moreover, more demonstrative signs and conditions such as headache, dizziness, myalgia, epileptic seizures, rhabdomyolysis and syndrome Guillain, anosmia, encephalitis, and insomnia after COVID-19 were documented [9,10,11,12].

These neurological symptoms in different combinations are referred to as cerebral/brain fog [13,14]. The time and form of manifestation of brain fog as the clinical condition may vary from cognitive problems, mostly short-term memory, attention impairment and problems with concentration [15] that were reported already during the onset of clinical SARS-CoV2 infection [16]. There is growing evidence, that brain fog represents organic sequelae of COVID-19 affecting the nervous system by means of chronic inflammatory processes in the nervous system [17,18] and disturbed neurotransmission [18]. Moreover, objective changes in brain metabolism, as documented by positron emission tomography (PET) have been described [19].

For this reason, several groups hypothesized, that COVID-19 infection may result—as an acute or chronic condition—in a change of electric brain activity, as recorded and documented by plain electroencephalography (EEG) or qualitative EEG. Indeed, some QEEG changes in post-COVID-19 patients were documented and linked with the recovery from psychopathological symptoms, including cognitive impairment [20]. Similar changes in EEG were described in mild cognitive impairment, not related to viral infection [21]. Moreover, in patients affected by COVID-19 in its more severe form, specific EEG disturbances were well documented [22]. Based on this observation, including EEG as the standard diagnostic and follow-up tool for COVID-19 encephalopathy [23] has been postulated. However, to date, no specific EEG or QEEG pattern related to brain fog as the less severe but still deteriorating sequel of COVID-19 infection has been delineated.

Based on this gap, as well as on the previous evidence of EEG changes in the COVID-19 course, we hypothesized that brain fog after COVID-19 may be characterized by a set of EEG changes, possibly enabling us to objectively confirm this diagnosis in subjects who self-report a cognitive disturbance. In order to analyze this topic, we performed qualitative EEG-based electrophysiologic analysis in a group of 20 patients demonstrating the symptoms of brain fog after COVID-19 infection as confirmed by a neurological and psychological workup. Importantly, QEEG records of the same individuals obtained prior to the COVID-19 infection were available enabling us to compare the QEEG pattern before and after the onset of the brain fog symptoms.

## 2. Materials and Methods

All study procedures were performed in accordance with relevant guidelines and regulations and after approval of the study protocol by the local Ethical Board.

### 2.1. Participants

A total of 20 people who revealed symptoms of COVID fog (10 men and 10 women) participated in our research. The study group consisted of people from the scientific community (with PhD), working mentally on a daily basis, aged between 36 and 45 years old.

### 2.2. Experimental Design

**Figure 1:**
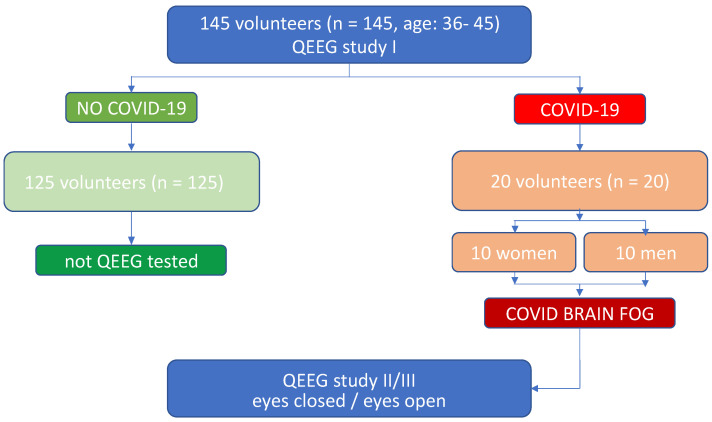
Experimental design.

In 2019, before the epidemic broke out, a QEEG workup was carried out among 145 employees of the University of Rzeszow, who decided to participate in the QEEG baseline screening test to learn about the QEEG method and to assess the activity of their own brain. Several months later, 20 people in this group were identified as having developed problems with memory, spatial orientation and concentration after contracting COVID-19. These people reported problems with remembering the content of the lecture materials. Therefore, after visiting a neurological clinic and performing a number of laboratory tests, as well as after obtaining a negative test for the persistence of coronavirus infection, the doctor proposed to perform a complementary QEEG diagnostic test. Due to technical and logistical availability to use QEEG according to the protocol used previously in a baseline screening, the option to perform the second QEEG test, being parallelly the follow-up of their electrophysiologic record appeared and the study participants after obtaining the full informed consent took advantage of it (Figure 1).

### 2.3. Measures for Identifying Brain Fog

First, an interview was conducted regarding the specificity of the symptoms. The selection of the main group was based on the occurrence of at least five symptoms that appeared after developing COVID-19, indicating cognitive problems. The subjects chose from the list the symptoms that they developed after their illness. These symptoms were as follows: problems with memory, frequent forgetting of words, problems with orientation in space, difficulties in organizing everyday activities, difficulties in interpersonal communication, forgetting what the previous speaker said, losing the thread in the discussion, feeling frequently tired, forgetting about many everyday matters, problems with concentration, insomnia, irritability, difficulty remembering events from a few days ago, feeling lost, difficulties in organizing activities and performing tasks at work. The symptom checklist has been developed for the specific purpose of the current study and encompasses the signs, which are provided by the previous literature as characteristic of the brain fog most frequently [15,24]. If the set of symptoms was assessed by the specialist in clinical neurology and/or by a clinical psychologist (external clinical workup) as sufficient to confirm the condition of post-COVID-19 brain fog, the subject was qualified as suitable for further participation in the study and subjected to the QEEG examination. This was carried out one month after receiving a negative coronavirus test.

### 2.4. QEEG Procedure

QEEG (quantitative electroencephalogram) is a numeric, spectral analysis of the EEG record, where the data is digitally coded and statistically analyzed using the Fourier transform algorithm [25,26]. Each examination of one person lasted about 10–15 min and included two stages: the first-recording of brain waves with eyes closed (3 min), the other with eyes open (3 min). The wave amplitude and power for specific frequencies were analyzed here. Taking into account the norms for adults, it is assumed that the lower the frequency of the waves, the lower the amplitude. Normal Delta waves below 20 µV, Theta below 15 µV, Alpha below 10 µV, sensimotor rhythm (SMR), Beta 1 and Beta 2: 4–10 µV according to the standard. The EEG signal was transformed using Cz montage and Cz electrode as the most common reference site [27] and by quantifying with the Elmiko, DigiTrack software (version 14, PL) (ELMIKO, Warsaw, Poland). Channels from the central lane were recorded. The study performed included Delta, Theta, Alpha, SMR, Beta 1, and Beta 2 waves at electrodes on the central lanes C3, C4. The amplitude of QEEG rhythms is calculated with medical standards of apparatus DigiTrack. The spectrum of a signal is a representation of this signal depending on the frequency. The algorithm FFT is used, with the result of the function: f(z) = A(z) + j*F(z). In FFT analysis, the following parameters have been implemented: minimal signal amplitude 0.5 µV with minimal temporal distance between single maximal values of 0.5 Hz. The analysis was provided using a computing buffer of 8.2 s (2048 assessment points, accuracy 0.12 Hz). As a result, the set of amplitude values for each defined part of the frequency spectrum has been obtained. The gap between single values, measured in Hz is defining a calculation resolution According to the FFT algorithm, this parameter depends on signal sampling frequency and on the length of the computing buffer,: r = fs/N, r—calculation resolution, i.e., the gap between single records, fs—signal sampling frequency, N—length of computing buffer. The results of spectrum analysis in the FFT panel in DigiTrack show amplitudes peak to peak. For the appropriate reliability, the measurement epochs of several seconds have been implemented [28]. The epoch length determines the frequency resolution of the Fourier, with a 1-second epoch providing a 1 Hz resolution (plus/minus 0.5 Hz resolution), and a 4-second epoch providing 0.25 Hz, or plus/minus 0.125 Hz resolution. The elimination of artifacts from the EEG recording was performed manually and automatically. Since the QEEG was intended as the basis for the potential subsequent QEEG-based neurofeedback intervention, the montage and channels most commonly used for the assessment and treatment of cognitive disturbances and sensimotor disintegration, i.e., central stripe electrodes (C3 and C4) were used for the further analysis [29].

### 2.5. Linking of Baseline to Experimental Subjects

Among the baseline records, those obtained in the study subjects before the onset of the brain fog symptoms were confidentially identified, retrieved and reliably allocated to the separate individuals. Thereafter, the newly obtained records were processed in the same manner. In that way, the pairs of measurements, obtained in the same individuals were created.

## 3. Statistical Analyses

The paired Wilcoxon test was used to compare two repeated measures of quantitative variables. The significance level for all statistical tests was set to 0.05. The results were presented as means ± SD. R 4.2.1. was used for computations. (R Core Team (2022). R: A language and environment for statistical computing. R Foundation for Statistical Computing, Vienna, Austria. URL: https://www.R-project.org/ (accessed on 22 April 2022)).

## 4. Results

The aim of the research was to compare the results of the QEEG study before and after the onset of COVID-19 and COVID-19-related brain fog symptoms. The qualitative analysis was performed separately for the eyes opened and eyes closed modus, as demonstrated in the graphic visualization of the data.

Taking into account the results of the Delta wave, in our exploratory experiment, there were no statistically significant changes in eyes open and eyes closed (Table 1).

The Theta wave’s amplitude from C3 and C4 channels was significantly affected by post-COVID-19 brain fog. After COVID-19 this parameter increased in right cerebral hemisphere by 26% (*p* < 0.001) in eyes open mode and 24% (*p* = 0.001) in eyes closed mode, respectively. In turn, in the left cerebral hemisphere, this increase was merely about 3% (*p* = 0.452) in eyes open mode but as high as and 40% (*p* < 0.001) in eyes closed mode (Table 2).

Similar to the Theta waves, the Alpha wave’s amplitude from C3 and C4 channels was also affected, when comparing this parameter before and after COVID-19 infection, again predominantly in the right hemisphere. In brain fog subjects, this parameter increased about 1.5% (*p* = 0. 0538), 5% (*p* = 0.042), 36% (*p* = 0.001), and 25% (*p* = 0.001) in the left (eyes open, eyes closed) and right (eyes open, eyes closed) cerebral hemisphere, respectively (Table 3).

Moreover, the SMR amplitude from C3 and C4 channels demonstrated changes after COVID-19 infection resulting in brain fog symptoms. After COVID-19 this parameter decreased about 26% (*p* < 0.001), 10.5% (*p* = 0.01), 8% (*p* = 0.017) in the left hemisphere (eyes open, eyes closed) and right (eyes closed) cerebral hemisphere, respectively. Only in the records from the right hemisphere (C4) with eyes open, a slight, non-significant increase in amplitude was observed (*p* = 0.332) (Table 4).

As to the Beta 1 wave’s amplitude, here the statistically significant differences were observed only in the left cerebral hemisphere. After COVID-19 this parameter increased about 17% (*p* = 0.001) and 8% (*p* = 0.014) in the C4-eyes open and closed, respectively (Table 5).

More prominent differences were seen in regard to the Beta 2 wave’s amplitude, here electrical activity of both the right and left hemispheres was significantly affected. After COVID-19 this parameter increased about 36% (*p* < 0.001) and 46% (*p* < 0.001) in the C3-eyes open and closed, respectively. Similarly, in the case of C4, an increase in the amplitude of about 70% (*p* < 0.001) and 49% (*p* < 0.001) was observed with eyes open and eyes closed, respectively (Table 6).

## 5. Discussion of Results and Conclusions

Our results demonstrate a clear difference in QEEG pattern in individuals suffering from brain fog symptoms after COVID-19 infection as compared with the baseline QEEG recorded before the onset of the disease. These are preliminary results of an exploratory study and certain caution is needed in their interpretation. In particular, a causative role of COVID-19 for observed impairment of electric brain activity may only be speculated and the possibility of the psychological load related to the pandemic situation rather than structural or functional changes resulting from (neuro-) infection by SARS-CoV2 needs to be considered. Nevertheless, this early observation confirms our main hypothesis, that brain fog symptoms may be accompanied by a change in QEEG pattern.

In our study, we attempted to document the post-COVID-19 related changes in patients with a confirmed diagnosis of brain fog using the QEEG approach. Our research shows that in the Delta wave range there was a decrease in the left hemisphere by 9%, and an increase in the right hemisphere by 7%. Such statistically significant differences were not observed in the pre-disease QEEG records. The study of Cecchetti et al. [20] also shows that people with the coronavirus had a lower Delta compared to healthy people. In our research in range of Theta frequencies, particularly pronounced changes after COVID-19 were found in the right hemisphere with eyes open, (*p* < 0.001) and in both C3 and C4 with eyes closed (*p* < 0.001 and *p* < 0.001). Similarly, in the study group the significant differences related to the Alpha amplitude were found in the right hemisphere in eyes closed (*p* < 0.001) while a significant increase in the amplitude was seen in the left hemisphere during the test in open and closed eyes, as compared to the records before COVID-19 (*p* = 0.001 and *p* < 0.01). Of note, Alpha and Theta variability and reactivity has been described by Pati S. et al. [30] as potential QEEG prognostic indicator in critically ill COVID-19 patients. Following, an EEG study performed by Vespignani et al. [22], among 26 patients with severe COVID-19-related symptoms, 19 of them displayed profound EEG disturbance characterized by dispersed and non-specific Theta-Alpha activities with dispersed Delta activity in some of them (here, non-focal and non-periodic pattern was documented). Similar results have been provided by van der Hiele K et al. [21] in subjects affected by non-COVID-19 related cognitive impairment. In their study, both Theta and Alpha reactivity were higher in subjects displaying symptoms of mild cognitive impairment. This pattern may be attributed to the symptoms of general fatigue and disturbed memory and concentration. A similar conclusion was provided by Li G. et al. [31] who assessed EEG changes during rest and physiological overload. In patients who were tasked with mathematical riddles of high difficulty level EEG was sampled before and during increased intellectual work. Here, Li G. et al. stated, that the relative power index of each EEG rhythm is more sensitive than the power index in response to mental fatigue, suggesting that relative power can be applied to estimate brain fatigue level. According to them, the relative power of each EEG rhythm is also better at assessing mental fatigue in a resting state than in a task state. The most important conclusion was that the Alpha frequency is the most relevant in fatigue assessment, as splitting the Alpha frequency band into the Alpha 1 band and Alpha 2 band may improve the sensitivity of the analysis. Of note, significant changes in the left hemisphere were also observed in the case of the Beta 1 wave amplitude. After COVID-19 this parameter increased (significant statistical differences in eyes open and closed, respectively: *p* = 0.001 and *p* = 0.014). A similar effect could be documented in the area of Beta 2 frequencies, with an increase in the amplitude observed in both hemispheres, when comparing this parameter before and after COVID-19 infection (in the C3 and C4, in eyes open and closed, respectively, *p* < 0.001). Another observation in our group was the drop in amplitude of sensimotor waves with a parallel increase in Beta 2 frequencies. In turn, with regard to SMR waves, COVID-19 and brain fog were related to SMR reduction, in relation to the records in groups before COVID-19, especially in the right hemisphere of the brain in eyes open. The differences observed after COVID-19 were statistically significant: *p* < 0.001). After COVID-19 SMR amplitude SMR in eyes closed decreased in C3 and C4 (*p* = 0.011 and *p* = 0.017). Similar effects on SMR and EEG spectrum have been documented also by Park et al. in their study on COVID-19 pandemic effects on EEG [32]. These changes resemble those documented recently by our own research group, where we indicated a change in the range of brain waves SMR in people suffering from generalized anxiety disorder (GAD) [33]. May be effects of psychological stress load due to diagnosed and treated COVID-19 need to be taken into account.

Summarizing, and based on our results, a specific pattern of QEEG changes in subjects affected by post-COVID-19 brain fog may be delineated:

1. Relative increase of Theta, Alpha and SMR frequencies in the right hemisphere as compared to the left hemisphere.
2. Remarkable increase in Beta 2 versus SMR in both hemispheres.
3. Increase in Beta 1 in the left hemisphere.
4. Reduction in SMR values

The described phenomena may result from several pathomechanims. Perhaps the disturbance in interhemispheric connectivity is caused by desynchronization of the peripheral autonomic system [34,35,36]. Other studies also confirm that among patients with COVID-19, the hemispheric connectivity is lower, in particular regarding asymmetric distribution for EEG bands in temporal lobes [3]. Significant differences in the activity of both cerebral hemispheres may be associated with disturbances in receiving and processing information. According to studies by other researchers, damage to the right hemisphere may affect both motor skills and emotional and cognitive processes, including memory problems [37,38]. Of note, much of information processing, including its storage (memory) is related to the emotional context of given information [39]. Thus, decreased emotion-related reactivity may hinder the process of remembering and associating.

In general, our analysis confirmed that in subjects with past COVID-19 infection and signs of who are claiming problems with memory, concentration, and thought disturbances, certain changes in EEG spectrum may be documented. A similar observation has been made by other research groups [23]. We believe, that QEEG may be useful in providing the objective documentation of otherwise subjective symptoms, possibly helping to attribute them to the brain fog at its beginning stage. Here, providing the objective result of the QEEG assessment may provide a certain relief for the subjects affected by brain fog symptoms concerned if their complaints are genuine or rather imaginary. The potential consequences of such prolonged uncertainty include a feeling of being lost, fear for one’s own health, decreased adaptation abilities, and most of all a feeling of helplessness, all of these potentially leading to even more serious mental disorders including depression and suicidality. Here, the QEEG assessment and its results may—at least partially—support the affected subjects about the realness and not illusory character of their complaints. Certainly, it would be premature to declare QEEG as a valid diagnostic tool based on the results of our exploratory study. However, this preliminary report urges the need for further, more systematic analysis of QEEG changes in subjects with chronic cognitive impairment after COVID-19. In case of adaptation of QEEG-based neurofeedback in therapeutic processes in these individuals, QEEG would enable us to monitor the changes in brain function during therapy of brain fog [40,41,42,43]. Considering the growing occurrence of post-COVID-19 brain fog, we believe, that the focus should be put on QEEG and QEEG-based biofeedback as having the potential to become an important diagnostic and therapeutic tool.

Certainly, as discussed above, the recorded changes may result not directly from the neurotrophic effect of the Sars-CoV2 virus, but also from the general psychological burden, related to the infection. Another problem is the deleterious effect of respiratory distress on neurologic function, including EEG records. Here, an overinterpretation of QEEG changes as a certain proof of direct brain affection by viral infection needs to be avoided. A reasonable approach would be a correlation analysis of virus burden with the EEG spectrum, ideally as a multivariate analysis, in order to refine the impact of the virus itself from systemic disease-related confounders [44]. Here, implementing QEEG instead of plain EEG records seems to be a valuable research and diagnostic concept [45].

Our study is not free from limitations. The major one is the low number of patients, subjected to our QEEG assessment. Here, we relied on the previous clinical diagnosis of post-COVID-19 brain fog. On the one hand, as a relatively new condition, this diagnosis is still rather reluctantly stated by clinical practitioners, thus limiting the size of the research group. On the other hand, due to such a skeptical, highly sensitive attitude, we are quite assured, that only the subjects with clear, full-blown brain fog were gated in our study. Moreover, the initial selection of our study group (academic researchers and teachers) warrants a certain homogeneity of individuals as to their primary intellectual capabilities. On the other hand, based on this preselection any overinterpretation of our data for the whole population of COVID-19-affected patients should be avoided. Another handicap of our analysis is the lack of a control group, recruited from the patients with no history of SARS-CoV2 infection. However, facing the fact of the high occurrence of COVID-19 infection in the general population (including the subset of highly SARS-CoV2 exposed academic teachers) and the risk of including patients with an asymptomatic course of the disease of the past, the creation of the homogenous, non-COVID-19 affected control group would be an extremely difficult task. We have circumnavigated this logistic obstacle by creating a pool of records serving as the intrinsic reference, composed of the QEEGs of all individuals included in the study before the COVID-19 pandemic outbreak. We believe that this unique possibility to assess the electrical brain activity of the very same subjects before and after the onset of post-COVID-19 symptoms was also the main advantage of our study. In this way, the impact of COVID-19 infection (regardless of its pathomechanism) could be documented with the pre-COVID-19 QEEG records serving as the intrinsic control group, personalized for each of the study individuals. Certainly, with previous pre-COVID-19 EEG records as the only control, our study was strongly reliant on the techniques and recordings implemented during the screening round of QEEG assessment. For this reason, more sophisticated methods of analysis such as CSD or IAF were not available for the set of data that were previously recorded and currently analyzed. Despite these drawbacks, we believe that our current study may fuel a discussion about the possible reasons for the QEEG changes observed by us and other research groups in post-COVID patients and that our results create an opportune starting point for further research focused on the full description of brain fog-associated QEEG pattern. Here, some more detailed analyses for further electrophysiological landmarks covering frontal and parietooccipital areas or using more specific EEG analysis algorithms are warranted.

## Figures and Tables

**Table 1:** The results of the Delta waves examination (waves from C3, C4 channels). The values are expressing the wave amplitude (in μV), with demonstration of main distribution parameters (including median and quartiles); *p*-values are referring to the results of the Wilcoxon test in a given set of records.

Mode		Pre-COVID	Post-COVID	*p*
C3, eyes open	mean ± SD	15.06 ± 0.83	13.58 ± 3.52	*p* = 0.073
	Median	15.11	12.85	
	Quartiles	14.46–15.5	10.46–16.61	
C4, eyes open	mean ± SD	15.49 ± 0.85	16.66 ± 3.84	*p* = 0.24
	Median	15.69	15.44	
	Quartiles	14.76–15.84	13.57–21.45	
C3, eyes closed	mean ± SD	14.35 ± 0.56	14.38 ± 3	*p* = 0.927
	Median	14.39	14.26	
	Quartiles	14.05–14.65	11.89–17.36	
C4, eyes closed	mean ± SD	14.94 ± 0.92	16.41 ± 3.21	*p* = 0.067
	Median	14.76	15.44	
	Quartiles	14.59–15.31	13.78–19.08	

p-ilcoxon paired test.

**Table 2:** The results of the Theta waves examination (waves from C3, C4 channels).

Mode		Pre-COVID	Post-COVID	*p*
C3, eyes open	mean ± SD	8.29 ± 0.59	8.55 ± 1.52	*p* = 0.452
	Median	8.36	9.06	
	Quartiles	7.7–8.78	7.02–9.87	
C4, eyes open	mean ± SD	8.49 ± 0.32	10.54 ± 1.93	*p* = 0.001 *
	Median	8.46	10.88	
	Quartiles	8.32–8.69	8.25–12.52	
C3, eyes closed	mean ± SD	7.59 ± 0.41	9.55 ± 0.98	*p* < 0.001 *
	Median	7.54	9.86	
	Quartiles	7.34–7.7	9.13–10.29	
C4, eyes closed	mean ± SD	7.89 ± 0.75	11.04 ± 1.41	*p* < 0.001 *
	Median	7.94	11.12	
	Quartiles	7.6–8.43	10.4–12.52	

p-Wilcoxon paired test. * statistically significant (*p* < 0.05).

**Table 3:** The results of the Alpha waves examination (waves from C3, C4 channels).

Mode		Pre-COVID	Post-COVID	*p*
C3, eyes open	mean ± SD	6.74 ± 0.74	6.84 ± 2.42	*p* = 0.538
	Median	7.08	6.42	
	Quartiles	6.19–7.18	6.06–6.61	
C4, eyes open	mean ± SD	6.58 ± 0.59	8.95 ± 2.64	*p* = 0.001 *
	Median	6.49	8.54	
	Quartiles	6.08–6.99	7.71–9.82	
C3, eyes closed	mean ± SD	6.02 ± 0.73	6.34 ± 0.35	*p* = 0.042 *
	Median	5.85	6.4	
	Quartiles	5.43–6.38	6.04–6.58	
C4, eyes closed	mean ± SD	6.36 ± 0.78	7.95 ± 1.31	*p* = 0.001 *
	Median	6.17	8.04	
	Quartiles	5.83–6.99	7.33–8.81	

p-Wilcoxon paired test. * statistically significant (*p* < 0.05).

**Table 4:** The results of the SMR waves examination (waves from C3, C4 channels).

Mode		Pre-COVID	Post-COVID	*p*
C3, eyes open	mean ± SD	4.33 ± 0.2	3.19 ± 0.23	*p* < 0.001 *
	Median	4.38	3.16	
	Quartiles	4.2–4.45	3.05–3.19	
C4, eyes open	mean ± SD	4.3 ± 0.33	4.53 ± 0.69	*p* = 0.332
	Median	4.23	4.48	
	Quartiles	4.05–4.4	4.23–4.61	
C3, eyes closed	mean ± SD	4.69 ± 0.64	4.2 ± 0.43	*p* = 0.011 *
	Median	4.44	4.16	
	Quartiles	4.28–4.99	4.05–4.19	
C4, eyes closed	mean ± SD	5.01 ± 0.64	4.63 ± 0.46	*p* = 0.017 *
	Median	4.85	4.56	
	Quartiles	4.4–5.73	4.31–4.73	

p-Wilcoxon paired test. * statistically significant (*p* < 0.05).

**Table 5:** The results of the Beta 1 waves examination (waves from C3, C4 channels).

Mode		Pre-COVID	Post-COVID	*p*
C3, eyes open	mean ± SD	4.53 ± 0.33	4.32 ± 0.52	*p* = 0.191
	Median	4.4	4.58	
	Quartiles	4.25–4.77	3.74–4.73	
C4, eyes open	mean ± SD	4.45 ± 0.33	5.23 ± 0.72	*p* = 0.001 *
	Median	4.46	5.36	
	Quartiles	4.39–4.53	4.47–5.68	
C3, eyes closed	mean ± SD	4.48 ± 0.28	4.53 ± 0.45	*p* = 0.823
	Median	4.36	4.71	
	Quartiles	4.25–4.61	4.47–4.76	
C4, eyes closed	mean ± SD	4.55 ± 0.29	4.93 ± 0.49	*p* = 0.014 *
	Median	4.51	5	
	Quartiles	4.45–4.62	4.47–5.39	

p-Wilcoxon paired test. * statistically significant (*p* < 0.05).

**Table 6:** The results of the Beta 2 waves examination (waves from C3, C4 channels).

Mode		Pre-COVID	Post-COVID	*p*
C3, eyes open	mean ± SD	5.01 ± 0.25	6.8 ± 1.08	*p* < 0.001 *
	Median	5.06	7.09	
	Quartiles	4.78–5.12	5.59–7.59	
C4, eyes open	mean ± SD	4.91 ± 0.58	8.33 ± 1.3	*p* < 0.001 *
	Median	4.64	8.7	
	Quartiles	4.38–5.43	6.69–8.94	
C3, eyes closed	mean ± SD	4.48 ± 0.53	6.54 ± 0.97	*p* < 0.001 *
	Median	4.4	6.59	
	Quartiles	4–4.94	5.59–7.5	
C4, eyes closed	mean ± SD	4.92 ± 0.62	7.33 ± 0.95	*p* < 0.001 *
	Median	4.97	7.08	
	Quartiles	4.36–5.43	6.69–7.74	

p-Wilcoxon paired test. * statistically significant (*p* < 0.05).

## Data Availability

The datasets generated during and/or analyzed during the current study are available from the corresponding author on reasonable request.
